# Baicalin reverses the impairment of synaptogenesis induced by dopamine burden via the stimulation of GABA_A_R–TrkB interaction in minimal hepatic encephalopathy

**DOI:** 10.1007/s00213-018-4833-8

**Published:** 2018-02-06

**Authors:** Saidan Ding, Weishan Zhuge, Jiangnan Hu, Jianjing Yang, Xuebao Wang, Fangfang Wen, Chengde Wang, Qichuan Zhuge

**Affiliations:** 1 Zhejiang Provincial Key Laboratory of Aging and Neurological Disease Research, The First Affiliated Hospital of Wenzhou Medical University, Wenzhou, Zhejiang, 325000 People’s Republic of China; 2 Gastrointestinal Surgery, The First Affiliated Hospital of Wenzhou Medical University, Wenzhou, Zhejiang, 325000 People’s Republic of China; 3 Institute for Healthy Aging, University of North Texas Health Science Center, Fort Worth, TX 76107 USA; 4 Zhejiang Provincial Key Laboratory of Aging and Neurological Disease Research, Neurosurgery Department, The First Affiliated Hospital of Wenzhou Medical University, Wenzhou, Zhejiang, 325000 People’s Republic of China; 5 School of Pharmaceutical Sciences, Wenzhou Medical University, Wenzhou, Zhejiang, 325000 People’s Republic of China

**Keywords:** Minimal hepatic encephalopathy, Dopamine, Baicalin, GABA_A_R, DARPP32

## Abstract

**Background:**

It has been reported that D1 receptor (D_1_R) activation reduces GABA_A_ receptor (GABA_A_R) current, and baicalin (BAI) displays therapeutic efficacy in diseases involving cognitive impairment.

**Methods:**

We investigated the mechanisms by which BAI could improve DA-induced minimal hepatic encephalopathy (MHE) using immunoblotting, immunofluorescence, and co-immunoprecipitation.

**Results:**

BAI did not induce toxicity on the primary cultured neurons. And no obvious toxicity of BAI to the brain was found in rats. DA activated D_1_R/dopamine and adenosine 3′5′-monophosphate-regulated phospho-protein (DARPP32) to reduce the GABA_A_R current; BAI treatment did not change the D_1_R/DARPP32 levels but blocked DA-induced reduction of GABA_A_R levels in primary cultured neurons. DA decreased the interaction of GABA_A_R with TrkB and the expression of downstream AKT, which was blocked by BAI treatment. Moreover, BAI reversed the decrease in the expression of GABA_A_R/TrkB/AKT and prevented the impairment of synaptogenesis and memory deficits in MHE rats.

**Conclusions:**

These results suggest that BAI has neuroprotective and synaptoprotective effects on MHE which are not related to upstream D_1_R/DARPP32 signaling, but to the targeting of downstream GABA_A_R signaling to TrkB.

## Introduction

Patients with minimal hepatic encephalopathy (MHE) show a complex neuropsychiatric syndrome including mild cognitive impairment, attention deficits, psychomotor slowing, and impaired bimanual and visuo-motor coordination, which can be unveiled using psychometric tests (Amodio et al. 2004; Montoliu et al. 2007). Our several previous studies showed that intracranial dopamine (DA) levels are abnormally elevated in MHE rats, and the elevation of intracranial DA plays an essential role in learning and memory impairment in MHE rats (Ding et al. 2013, 2014a, b, c). The pathogenesis of MHE has been confirmed to be caused by the elevation of DA (Ding et al. 2013). Although DA is known to modulate voltage-dependent ion channels in neostriatal neurons (Surmeier et al. 1995), elevated DA levels have been implicated in several neurological and psychiatric disorders, including schizophrenia, Huntington’s disease, Alzheimer’s disease, attention deficit disorder, and addictive behavior (Blum et al. 2012; Ungless and Grace 2012). However, the deeper mechanism underlying the effects of DA on the pathogenesis of MHE remains unclear, and therapeutic drugs for MHE are lacking.

The toxic actions of DA appear to involve several distinct mechanisms, including autoxidation, oxidative stress, and D1 receptor activation (Chen et al. 2004). Importantly, DA released by midbrain dopaminergic neurons in both the striatum and the substantia nigra (Cheramy et al. 1981; Geffen et al. 1976) controls g-aminobutyric acid (GABA) release through the activation of D1 receptors (D1Rs) located at the somato-dendrites and terminals of striato-nigral medium spiny neurons (Acosta-Garcia et al. 2009). A variety of biochemical studies as well as targeted deletion and mutation of dopamine and adenosine 3′5′-monophosphate-regulated phospho-protein (DARPP32) in mice have shown that DARPP32 plays a critical role in the actions of DA (Svenningsson et al. 2003). Mice lacking DARPP32 exhibit profound deficits in their molecular, electrophysiological, and behavioral responses to DA (Greengard et al. 1999). D_1_R stimulation in neostriatal medium spiny neurons reduces postsynaptic GABA_A_ receptor currents by activating a PKA/dopamine- and adenosine 3′5′-monophosphate-regulated DARPP32/protein phosphatase 1 signaling cascade targeting GABA_A_ receptor β1 subunits (Flores-Hernandez et al. 2000). However, we hypothesized that the mechanisms of cognitive loss are related to the action of D1R/DARPP32/GABA_A_R signaling in MHE.

Baicalin (7-d-glucuronic acid-5,6-dihydroxyflavone; BAI) is a flavone isolated from the Chinese medicinal herb *Scutellaria baicalensis* Georgi that has been used to treat inflammatory diseases and ischemic stroke for thousands of years in China (Zhang et al. 2006). BAI has been identified as a novel and promising therapeutic agent for human central nervous system diseases, because it can pass through the blood–brain barrier into the central nervous system and distribute within the brain tissue, specifically in the hippocampus, striatum, cortex, and thalamus (Tarragó et al. 2008). In order to establish the optimal therapeutic usage of BAI, its physiological effects need to be thoroughly elucidated. The neuroprotective effects of BAI have been described in many experimental models, such as cerebral ischemia (Cheng et al. 2013; Zhou et al. 2014), spinal cord injury (Cao et al. 2010), epilepsy (Liu et al. 2012), learning and memory deficits (Lee et al. 2014), and cultured neurons (Yin et al. 2011). We hypothesized that BAI protects against memory decline in MHE.

Baicalin, along with its aglycone baicalein, is a positive allosteric modulator of the benzodiazepine site and/or non-benzodiazepine site of the GABAA receptor (Hui et al. 2000). Recent studies further demonstrated the anxiolytic-like effect of BAI in a Vogel conflict test and an elevated plus maze test and suggested that its pharmacological action occurred through GABA_A_ receptors (Xu et al. 2006). BAI may inhibit D1R-stimulated GABA_A_R suppression. However, the neuroprotective effects of BAI in the MHE rat brain may be related to the suppression of DA-induced GABA_A_R inhibition. However, there is little information about the therapeutic effects of BAI on DA-induced toxicity in vivo. Here, we tested the efficacy of BAI in MHE rats and in a DA injection rat model and investigated the potential mechanisms of its function.

The present study investigated the hypothesis that BAI protects against MHE and that the neuroprotective effects are associated with the activation of GABA_A_R, the modulation of downstream tropomyosin receptor kinase B (TrkB) signaling, and the expression of synapse-related proteins. The study focused on the evaluation of the effects of BAI on MHE and explored the potential molecular mechanisms using MHE and DA-injected rats.

## Materials and methods

### Primary hippocampal and cortical neuron cultures and treatments

Primary hippocampal neurons (PHNs) or primary hippocampal neurons (PCNs) were prepared from 1-day-old Sprague–Dawley rat pups. PHNs or PCNs were dissociated from the freshly dissected hippocampus or cerebral cortex by mechanical disruption in the presence of trypsin and DNase and then plated in poly-l-lysine-precoated six-well plates. Cells were seeded at a density of 2 × 10^6^ cells per well in a Neurobasal Medium (1X) supplemented with 0.5 mM GlutaMAX™-I, B-27® incubated at 37 °C, 5% CO_2_. The medium was changed after 4 days. Neurons remained untreated or were stimulated with DA (10 μM) in the presence or absence of BAI (1, 2.5, 5, 10, or 30 μM). PHNs transfected with TrkB siRNA were treated with DA (10 μM) in the presence or absence of BAI.

### Gene and siRNA transfection to PCNs

PCNs were transfected with 0.25 μg of TrkB siRNA (Santa Cruz, CA, USA) using Lipofectamine 2000 (Invitrogen) according to the protocol suggested by the manufacturer. The final concentration of siRNA was 10 nM. The Silencer Negative Control No. 1 siRNA (scrambled siRNA) was used as a control (Santa Cruz, CA, USA). After 48 h, cells were washed with Hank’s Balanced Salt Solution and treated with DA in the presence or absence of BAI.

### MHE models

Sprague–Dawley rats (Experimental Animal Center of the Chinese Academy of Sciences in Shanghai) weighing 220–250 g were used. Rats were housed under controlled temperature (24 ± 1 °C) and light (12 h light starting at 07:00 a.m.) conditions, and all experiments were carried out in accordance with the guidelines laid down by the Ethics Committees of the Affiliated Hospital of Wenzhou Medical University regarding the care and use of animals for experimental procedures (Albrecht et al. 2000).

Before experimenting, all animals underwent two behavioral tests: the Y-maze (YM) and the water-finding task (WFT). The normal values of these behavioral tests were obtained. Rats (*n* = 55) were then randomly divided into two groups: the control group (*n* = 10) and the thioacetamid (TAA) group (*n* = 45). Liver cirrhosis was induced by intraperitoneal (IP) injection of TAA (200 mg/kg in normal saline, Sigma-Aldrich) twice per week for 8 weeks. TAA-treated rats with symptoms were diagnosed with HE according to the following criteria: delayed development of decreased motor activity, lethargy, and eventual progression to coma. TAA-treated rats with no HE symptoms were then again subjected to behavioral tests to confirm whether or not they had MHE. If TAA-treated rats met either of the following criteria, they were included in the MHE group: (a) YM value lower than mean ± 1.96·SD, (b) WFT value greater than mean ± 1.96·SD.

WT and MHE rats were injected intraperitoneally with BAI (20, 50, or 100 mg/kg) three times per week for 1 week. At 24 h after the last injection, rats were subjected to YM and WFT tests. Then, all rats were sacrificed to collect the blood, liver tissues, and cerebral cortex tissues.

### DA-treated rat models and treatments

Rats (*n* = 40) were randomly divided into two groups: a DA-treated group (*n* = 30) and a control group (n = 10). Rats were anesthetized with an intraperitoneal injection of 10% chloralhydrate (0.4 g/kg). The rats were then placed on a stereotaxic apparatus. An intracerebroventricular injection of dopamine hydrochloride (10 μg/3 μl in saline) was stereotaxically injected in the left lateral ventricles of rats three times at 7-day intervals (anterior–posterior, 0.3 mm; lateral, 1.0 mm; horizontal, 3.0 mm from the bregma; *n* = 30). Then, DA-treated rats were intraperitoneally injected with BAI (20, 50, or 100 mg/kg) three times in 1 week. At 24 h after the last injection, rats underwent YM and WFT tests and were then killed to collect the blood, liver tissues, and cerebral cortex tissues.

### Y-maze test

Rats were individually placed at the end of an arm and allowed to explore the maze freely for 8 min. Spontaneous alternation percentage (SA%) was defined as the ratio of the arm choices that differed from the previous two choices (“successful choices”) to the total choices during the run (“total entry minus 2” because the first two entries could not be evaluated) (Yamada et al. 2005).

### Water-finding task

A rat was placed at the near-right corner of the apparatus and allowed to explore it freely for 3 min. The elapsed times until the entry into the alcove (entry latency (EL)), until the touching/sniffing/licking of the water tube (contacting latency (CL)), and until the initiation of drinking from the water tube (drinking latency (DL)) were measured (Kawasumi et al. 2004).

### Co-immunoprecipitations

Lysates were centrifuged, and supernatants were incubated with antibodies overnight (4 °C) and subsequently incubated with protein G agarose beads (Millipore) for 5 h (4 °C). Beads were washed three times with lysis buffer, and immunoprecipitated proteins were resolved by SDS-PAGE. Following transfer, proteins were probed using primary antibodies and secondary antibodies. Data are expressed as fold change relative to control for three or four independent experiments.

### MTT assay

Cells were incubated with medium containing 0.5 mg/ml MTT at 37 °C for 3 h and then treated with dimethylsulfoxide. The absorbance of each aliquot at 490 nm was determined using a microplate reader (Tecan). Cell viability was expressed as the ratio of the signals obtained from the treated and control cultures.

### Semi-quantitative PCR

Total RNA was extracted from cerebral cortex tissues or PCAs using TRIzol Reagent (Invitrogen). First-strand cDNA was synthesized from 1 μg total RNA using Omniscript Reverse Transcriptase (QIAGEN). PCR amplification with *Taq* DNA polymerase (Sigma-Aldrich) was performed under denaturation at 94 °C for 30 s, annealing at 60 °C for 30 s, and elongation at 72 °C for 90 s, repeating the indicated cycles. The sequences for the forward and reverse primers are as follows: GABA_A_R β1, forward 5’-CCGGCAAGGGGCGCA-3’ and reverse 5’-TCAGTCAAGTCGGGGATCTTCACT-3’; GABA_A_R β3, forward 5’-AGAGCATGCCCAAGGAAGG-3’ and reverse 5’-AGGTGGGTCTTCTTGTGCG-3’; TrkB, forward 5’-CGGAATTCATGTCGCCCTGGCCGAGGTG-3’ and reverse 5’-CGGGATCCCAGCCTTGTCTTTCCTTTATCT-3’; PSD95, forward 5’-CAAAGACCGTGCCAACGAT-3’ and reverse 5’-GGGACACAGGATCCAAACTTGT-3’; synapsin I, forward 5’-TTCAGCATGGCACGTAATGG-3’ and reverse 5’-CCAGCATACTGCAGCCCAAT-3’; GAPDH, forward 5’-ACCCAGAAGACTGTGGATGG-3’ and reverse 5’-ACACATTGGGGGTAGGAACA-3’.

### Immunoblotting

The total amount of protein in the lysates was determined by BCA protein assay (AMRESCO). Samples (50 μg protein) were separated by 10% SDS-PAGE and electroblotted to PVDF membranes, which were blocked by incubation in 5% non-fat dry milk dissolved in TBS-T (150 mM NaCl, 50 mM Tris, 0.05% Tween 20). Following transfer, proteins were probed using the following primary antibodies: D_1_R, DARPP32, GABA_A_Rβ1, GABA_A_Rβ3, TrkB, pAKT, AKT, synaptotagmin, synapsin I, PSD95, spinophilin, and β-actin (Abcam). Then, horseradish peroxidase-conjugated secondary antibody was used. After extensive washing, protein bands detected by antibodies were visualized by ECL reagent (Thermo) after exposure on Kodak BioMax film (Kodak).

### Double-labeled fluorescent staining

Four-micron frozen cerebral cortex sections or PCAs cultured on glass coverslips were fixed with 4% paraformaldehyde for 30 min and then treated with 0.1% Triton X-100 for 10 min at room temperature. Blocking was achieved with PBS containing 5% normal goat serum for 1 h at room temperature. Sections were then incubated overnight at 4 °C with the following primary antibodies: GABA_A_Rβ1, GABA_A_Rβ3, TrkB, pAKT, synapsin I, PSD95, GAP43, VGAT, bassoon, and MAP2 (Abcam). Binding of primary antibodies was detected by incubating the sections for 30 min with FITC (green)/Alexa Fluor 594 (red)-conjugated secondary antibody. Imaging was performed with a Leica TCS SP2 confocal laser scanning microscope. For the analysis of synaptogenesis in PHNs, the neurons were co-stained with anti-VGAT and anti-bassoon antibodies. The number and size of the synapses were analyzed with the ImageJ software.

### Electrophysiological analysis

Rats were anesthetized with isoflurane and decapitated, and the hippocampi were cut into 400-mm thick transverse slices using a vibratome. After incubation in artificial cerebrospinal fluid (aCSF) at room temperature for 60–90 min, slices were placed in a recording chamber on the stage of an upright microscope (Olympus CX-31) and perfused with aCSF (containing 1 mM MgCl_2_) at a rate of 3 ml per min at 23–24 °C. A 0.1 MΩ tungsten monopolar electrode was used to stimulate the Schaffer collaterals. The field excitatory postsynaptic potentials (fEPSPs) were recorded in CA1 stratum radiatum by a glass microelectrode filled with aCSF, with resistance of 3–4 MΩ. Field potential input–output curves were constructed by measuring the fEPSP slope corresponding to the stimulus intensity increase from 1 to 7 V in 0.5 V increments. A long-term potentiation (LTP) of fEPSPs was induced by three theta-burst stimulation (TBS), consisting of four pulses at 100 Hz repeated three times at 200-ms intervals. Paired-pulse facilitation (PPF) was examined by applying pairs of pulses at 20–500 ms intervals. The magnitude of LTP is expressed as the mean percentage of the initial baseline fEPSP slope.

### Statistical analysis

Data are presented as mean ± SD. The statistical significance of between-group comparisons was determined by one-way analysis of variance (ANOVA). Values of *P* < 0.05 or *P* < 0.01 were considered to be statistically significant.

## Results

### BAI has no effect on DA-mediated DARPP32 signaling in vitro

**Fig. 1:**
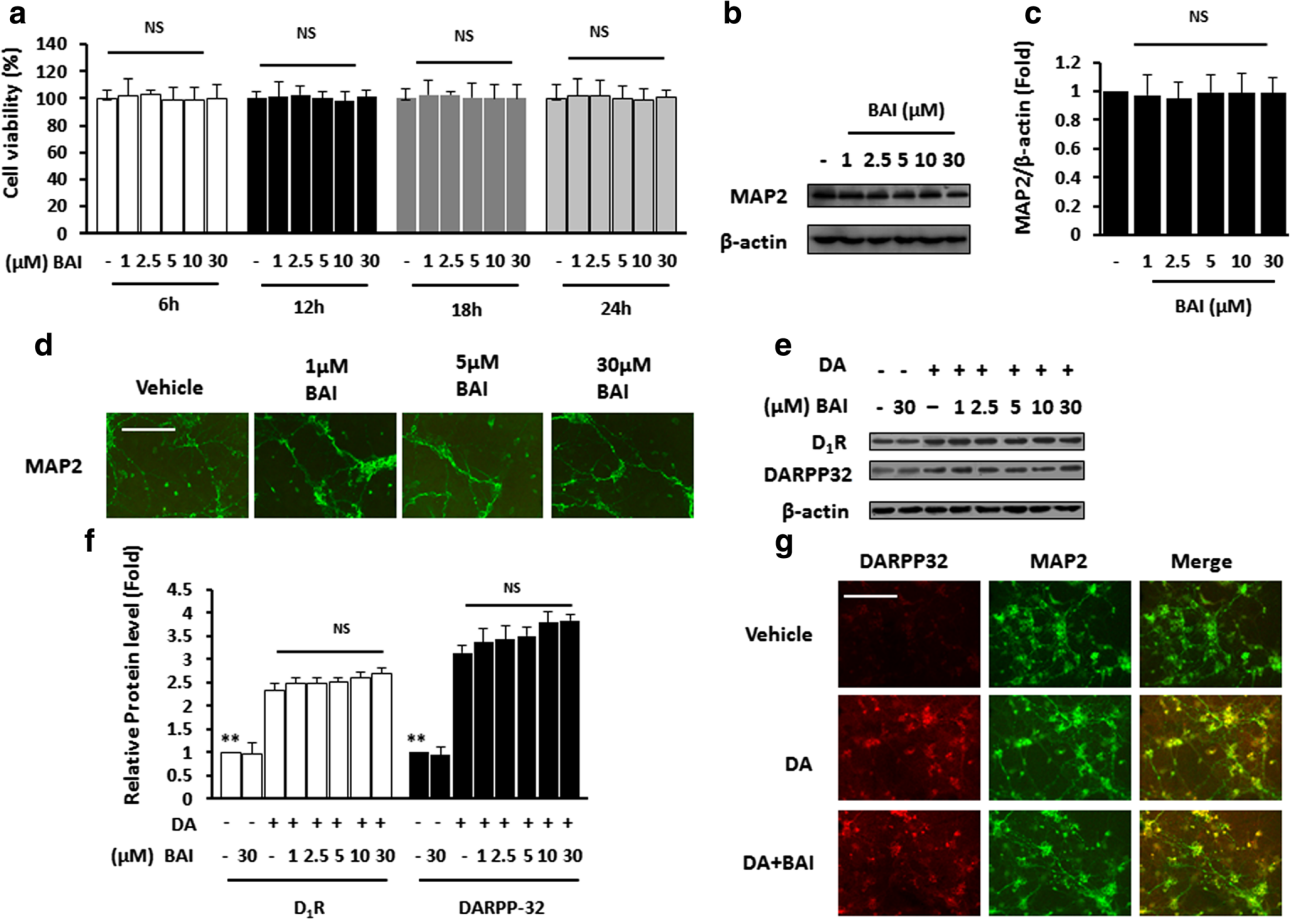
BAI has no effect on DA-mediated DARPP32 signaling in vitro. **a** MTT results for PHNs stimulated with 1–30 μM BAI at different time points. **b** Immunoblot analysis of lysates of PHNs exposed to various doses of BAI (1–30 μM) using anti-MAP2 and anti-β-actin antibodies and **c** subsequent densitometry. **d** Immunofluorescence staining of PHNs in the presence of various doses of BAI (1–30 μM) using antibodies against MAP2 (green). **e** Immunoblot analysis of lysates of PHNs stimulated with 10 μM DA in the presence of various doses of BAI (1–30 μM) using anti-D_1_R/DARPP32 and anti-β-actin antibodies and **f** subsequent densitometry. **g** Double immunofluorescence staining of PHNs stimulated with 10 μM DA in the presence of various doses of BAI (1–30 μM) using antibodies against DARPP32 (red), MAP2 (green). NS not significant. **P < 0.01 vs. DA-treated PCNs. Scale bar, 25 μm. MRGD, merged image

The potential toxicity of BAI to neurons had never been evaluated prior to this study. We first examined the effect of BAI on cell viability. The MTT assay showed that 0–30 μM BAI did not result in any cell death or significant effects on the cell viability of the PHNs (Fig. 1a). No obvious differences in the expression of MAP2 were found between the vehicle- and BAI-treated PHNs (Fig. 1b, c). MAP2 immunostaining revealed no obvious differences in the distribution or intensity of the immunofluorescence between the vehicle- and the BAI-treated PHNs (Fig. 1d). These results suggest that BAI has no toxic effect on neurons in vitro.

It has been reported that D_1_R stimulation in neostriatal medium spiny neurons reduces postsynaptic GABA_A_ receptor currents, and GABA_A_R activation triggers synaptic scaling (Wenner 2014). Since BAI is known as a modulator of GABA_A_R activation (Flores-Hernandez et al. 2000), we aimed to examine whether BAI enhanced the effect of DA on DARPP32 signaling. Immunoblotting from cell lysates showed that increasing concentrations of DA significantly increased the expression of D_1_R and DARPP32, but GABA_A_R activation by BAI had no effect on D_1_R or DARPP32 levels (Fig. 1e, f). Immunostaining showed increases in cell-bound DARPP32 in rat PCNs exposed to DA. The addition of BAI did not impact DA-induced increases in cell-bound DARPP32 (Fig. 1g).

### BAI inhibits the DA-induced inactivation of GABA_A_R in vitro

**Fig. 2:**
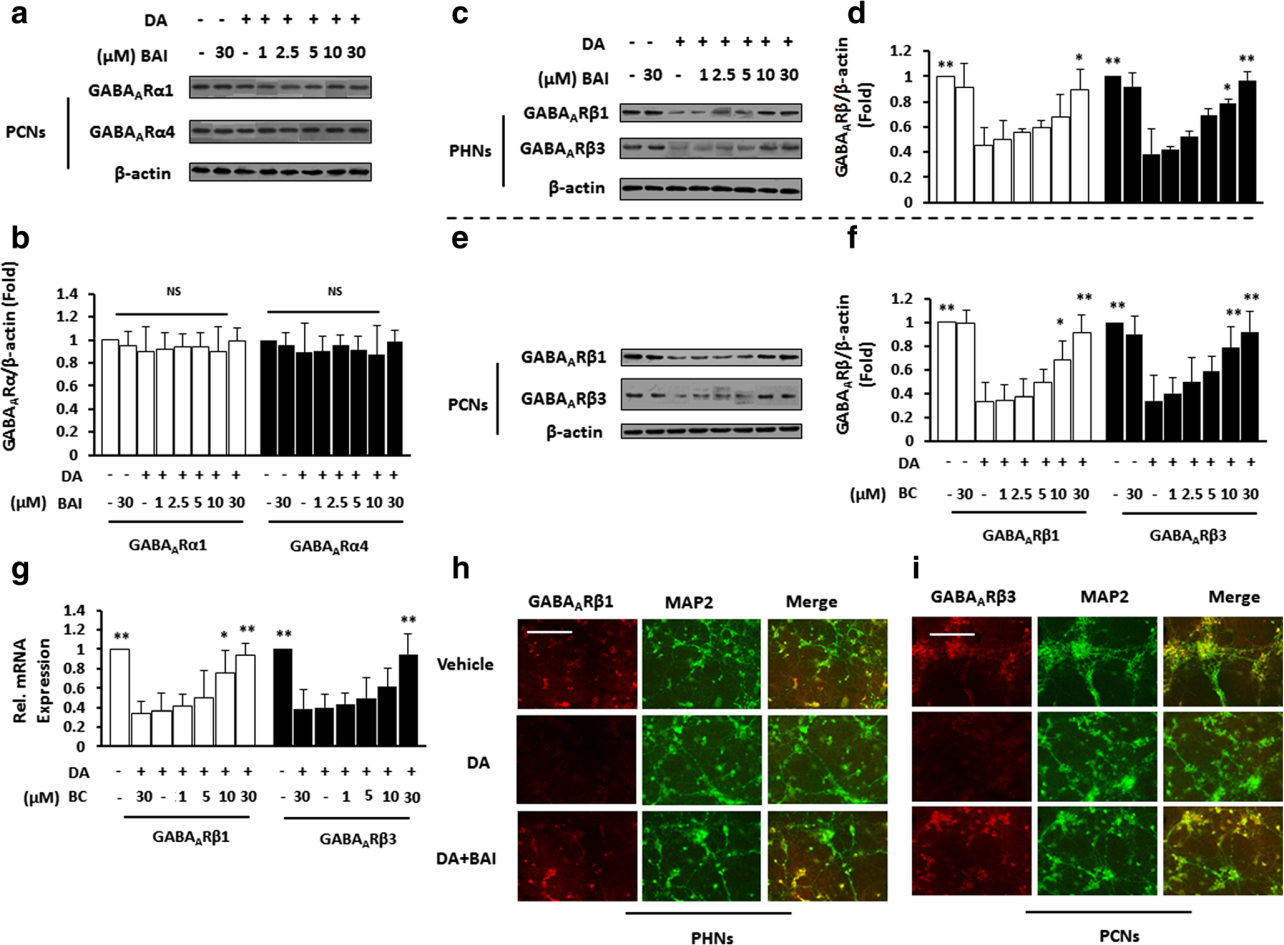
BAI reverses DA-induced inactivation of GABA_A_R. **a** Immunoblot analysis of lysates of PHNs stimulated with 10 μM DA in the presence of various doses of BAI (1–30 μM) using anti-GABA_A_Rα1/GABA_A_Rα2 and anti-β-actin antibodies and **b** subsequent densitometry. **c**–**f** Immunoblot analysis and densitometry of PHNs (**c**, **d**) and PCNs (**e**, **f**) stimulated with 10 μM DA in the presence of various doses of BAI (1–30 μM) using anti-GABA_A_Rβ1/GABA_A_Rβ3 and anti-β-actin antibodies. **g** GABA_A_Rβ1/GABA_A_Rβ3 mRNA in PCNs exposed to 10 μM DA in the presence of various doses of BAI (1–30 μM) were monitored by qPCR. **h** Double immunofluorescence staining of PHNs stimulated with 10 μM DA in the presence of BAI (30 μM) using antibodies against GABA_A_Rβ1 (red), MAP2 (green). Scale bar, 25 μm. MRGD, merged image. **i** Double immunofluorescence staining of PCNs stimulated with 10 μM DA in the presence of BAI (30 μM) using antibodies against GABA_A_Rβ 3 (red), MAP2 (green). *P < 0.05, **P < 0.01 vs. DA-treated PCNs. Scale bar, 25 μm. MRGD, merged image

TrkB signaling has a direct role in the formation and maintenance of synapses (Hiester et al. 2013). We therefore examined whether BAI triggered GABA_A_R to interact with TrkB. IB analysis showed no obvious differences in the expression of GABA_A_Rα between the vehicle- and the DA-treated PHNs. The addition of 0–30 μM BAI also had no significant effect on the expression of GABA_A_Rα (Fig. 2a, b).

IB analysis showed that DA significantly decreased GABA_A_Rβ1 and GABA_A_Rβ3 expression in PHNs compared with unstimulated cells. Treatment with BAI abolished the effect of DA on the expression of GABA_A_Rβ in a dose-dependent manner (Fig. 2c, d). In PCNs, DA treatment decreased the expression of GABA_A_Rβ1 and GABA_A_Rβ3, and the addition of BAI abrogated the effect of DA on GABA_A_Rβ1 and GABA_A_Rβ3 expression in a dose-dependent manner (Fig. 2e, f). QPCR showed that DA decreased GABA_A_Rβ1 and GABA_A_Rβ3 mRNA, and BAI treatment enhanced this effect in a dose-dependent manner in PHNs (Fig. 2g). Immunostaining further confirmed that DA significantly increased the expression of GABA_A_Rβ1 in PHNs (Fig. 2h) and GABA_A_Rβ3 in PCNs (Fig. 2i). BAI abolished the DA-stimulated reduction of GABA_A_Rβ levels. These results suggest that both DA and BAI do not affect the activation of GABA_A_Rα but induce the activation of GABA_A_Rβ.

### BAI improves the interaction of GABA_A_R with TrkB and downstream signaling in DA-treated neurons

**Fig. 3:**
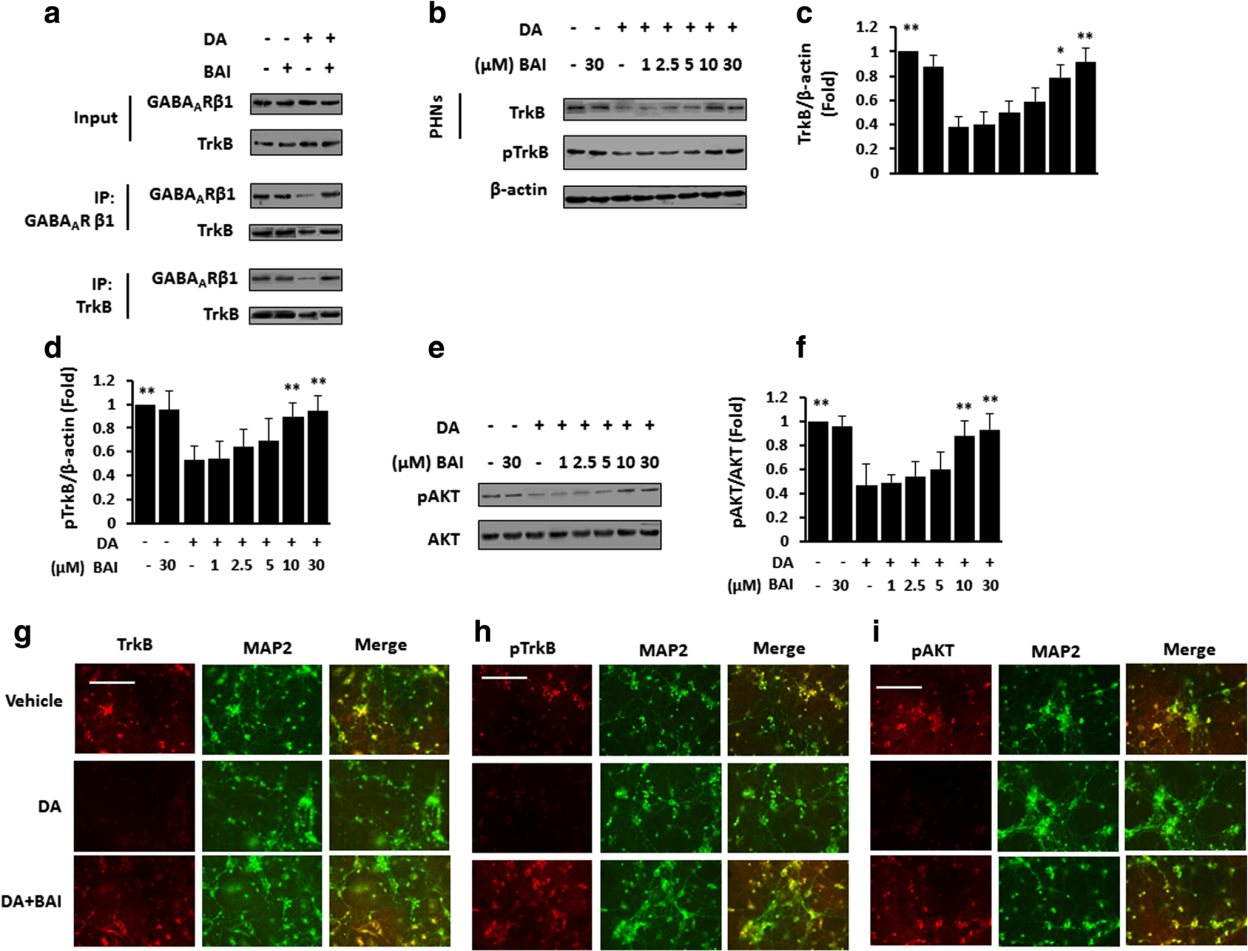
BAI reverses DA-induced disruption of the interaction of GABA_A_R with TrkB and downstream signaling inactivation in vitro. **a** Immunoprecipitation of lysates from PHNs stimulated with 10 μM DA in the presence of BAI (30 μM) with control IgG, anti-TrkB, or anti-GABA_A_Rβ1 antibodies. Complexes were immunoblotted with anti-TrkB, or anti-GABA_A_Rβ1 antibodies. **b** Immunoblot analysis and densitometry of PHNs stimulated with 10 μM DA in the presence of various doses of BAI (1–30 μM) using anti-TrkB/pTrkB and anti-β-actin antibodies and subsequent densitometry (**c**, **d**). **e** Immunoblot analysis of PHNs stimulated with 10 μM DA in the presence of various doses of BAI (1–30 μM) using anti-pAKT and anti-AKT antibodies and subsequent densitometry (**f**). **g**–**i** Double immunofluorescence staining of PHNs stimulated with 10 μM DA in the presence of BAI (30 μM) using antibodies against TrkB (g)/pTrkB (**h**)/pAKT (**i**) (red), MAP2 (green). *P < 0.05, **P < 0.01 vs. DA-treated PCNs. Scale bar, 25 μm. MRGD, merged image

We examined the effect of BAI on the DA-mediated interaction of GABA_A_Rβ and TrkB by co-immunoprecipitation. Then, lysates of PHNs were immunoprecipitated with a GABA_A_ARβ antibody; as predicted, DA induced a decrease in GABA_A_Rβ and also decreased the amount of TrkB that co-immunoprecipitated with GABA_A_Rβ, which was blocked by BAI. When lysates of PHNs were immunoprecipitated with TrkB antibody, as predicted, DA also decreased the amount of GABA_A_Rβ that co-immunoprecipitated with TrkB and increased the level of TrkB, which was also blocked by BAI (Fig. 3a). Immunoblotting confirmed that DA decreased TrkB expression and BAI treatment enhanced this effect in a dose-dependent manner in PHNs (Fig. 3b, c); additionally, DA decreased the phosphorylation of TrkB, and BAI treatment enhanced the DA-induced decrease in phosphorylated TrkB (pTrkB) in a dose-dependent manner in PHNs (Fig. 3b, d). Moreover, DA decreased the phosphorylation of AKT, and BAI enhanced this effect in a dose-dependent manner in PCNs (Fig. 3e, f). IF analysis confirmed that TrkB (Fig. 3g), pTrkB (Fig. 3h), and pAKT (Fig. 3i) were strongly expressed in the DA-treated PHNs, while BAI reduced the expression of TrkB/pTrkB/pAKT. These data suggest that BAI inhibits the DA-induced inactivation of the GABA_A_Rβ/TrkB signaling pathway in neurons.

### BAI prevents DA-induced impairment of synaptogenesis in vitro

**Fig. 4:**
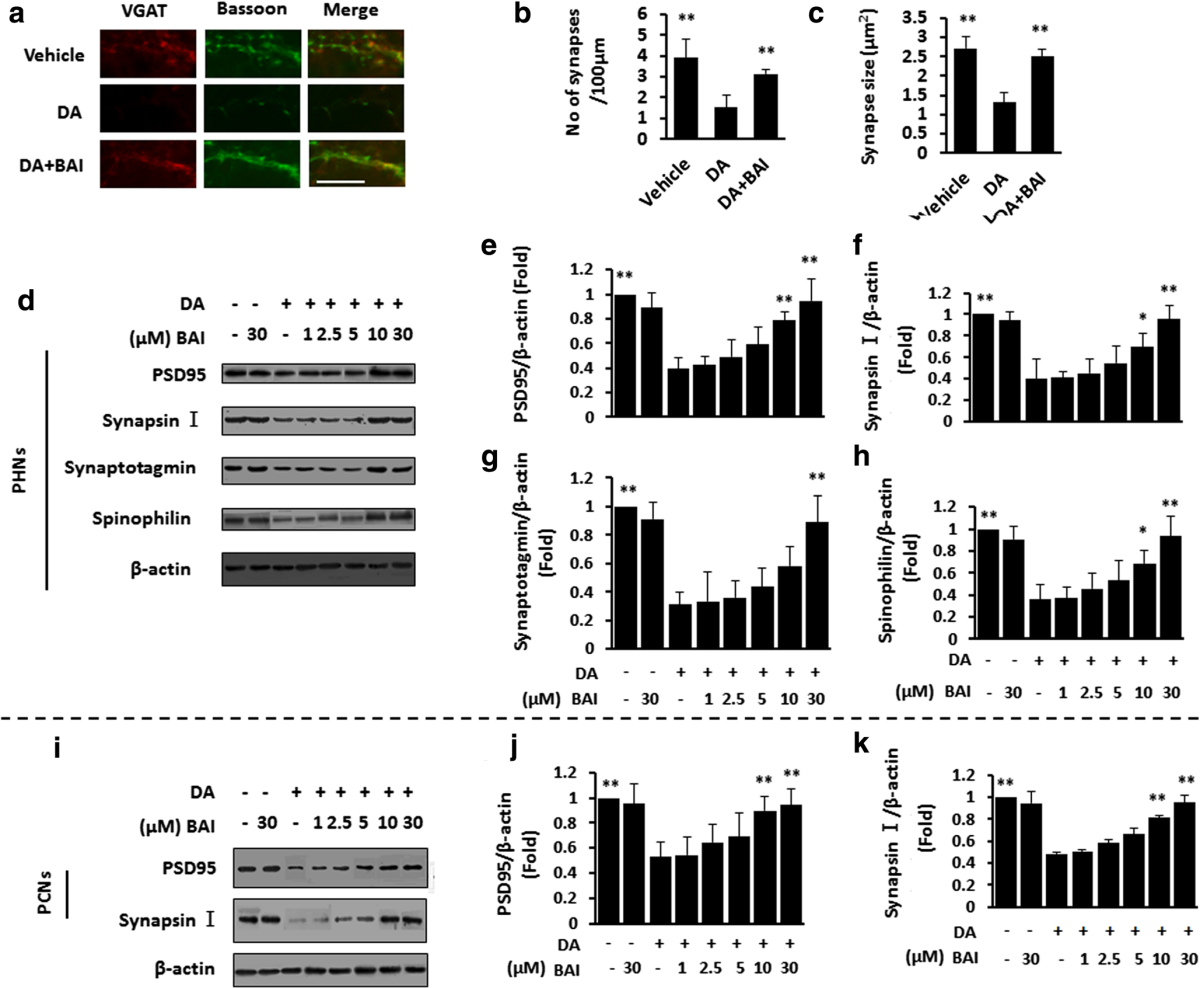
BAI prevents DA-induced impairment of synaptogenesis in vitro. **a** Double immunofluorescence staining of PHNs stimulated with 10 μM DA in the presence of BAI (30 μM) using the presynaptic markers VGAT antibody (green) and bassoon antibody (red). The number of synapses (**b**) and synapse size (**c**) were quantified. (**d**) Immunoblot analysis of PHNs stimulated with 10 μM DA in the presence of various doses of BAI (1–30 μM) using anti-PSD95/synapsin/synaptotagmin/spinophilin and anti-β-actin antibodies and subsequent densitometry (**e**–**h**). *P < 0.05, **P < 0.01 vs. vehicle-treated PCNs. **i** Immunoblot analysis of PCNs stimulated with 10 μM DA in the presence of various doses of BAI (1–30 μM) using anti-PSD95/synapsin I and anti-β-actin antibodies and subsequent densitometry (**j**). **k** PSD95/synapsin I mRNA in PHNs exposed to 10 μM DA in the presence of various doses of BAI (1–30 μM) were monitored by qPCR. * treated with DA in the presence or absence of BAI. *P < 0.05, **P < 0.01 vs. DA-treated PCNs. Scale bar, 25 μm. MRGD, merged image

Next, we examined the effect of BAI on the DA-induced impairment of synaptic formation. For the analysis of synaptogenesis in primary hippocampal neurons, the neurons were co-stained with anti-VGAT and anti-bassoon antibodies. The results showed that DA treatment decreased the number of presynaptic structures expressing VGAT and bassoon, and the addition of BAI reversed this effect (Fig. 4a–c).

IB analysis showed that DA treatment decreased the expression of PSD95/synapsin I/synaptotagmin/spinophilin, and the addition of BAI to the DA mixture nearly restored the four proteins to the basal level in PHNs (Fig. 4d–h). We confirmed that PSD95 and synapsin I were weakly expressed in response to DA and strongly expressed in response to BAI in PCNs (Fig. 4i, j). QPCR showed decreased PSD95/synapsin I mRNA in PHNs in response to DA, and the addition of BAI abrogated this effect (Fig. 4k).

**Fig. 5:**
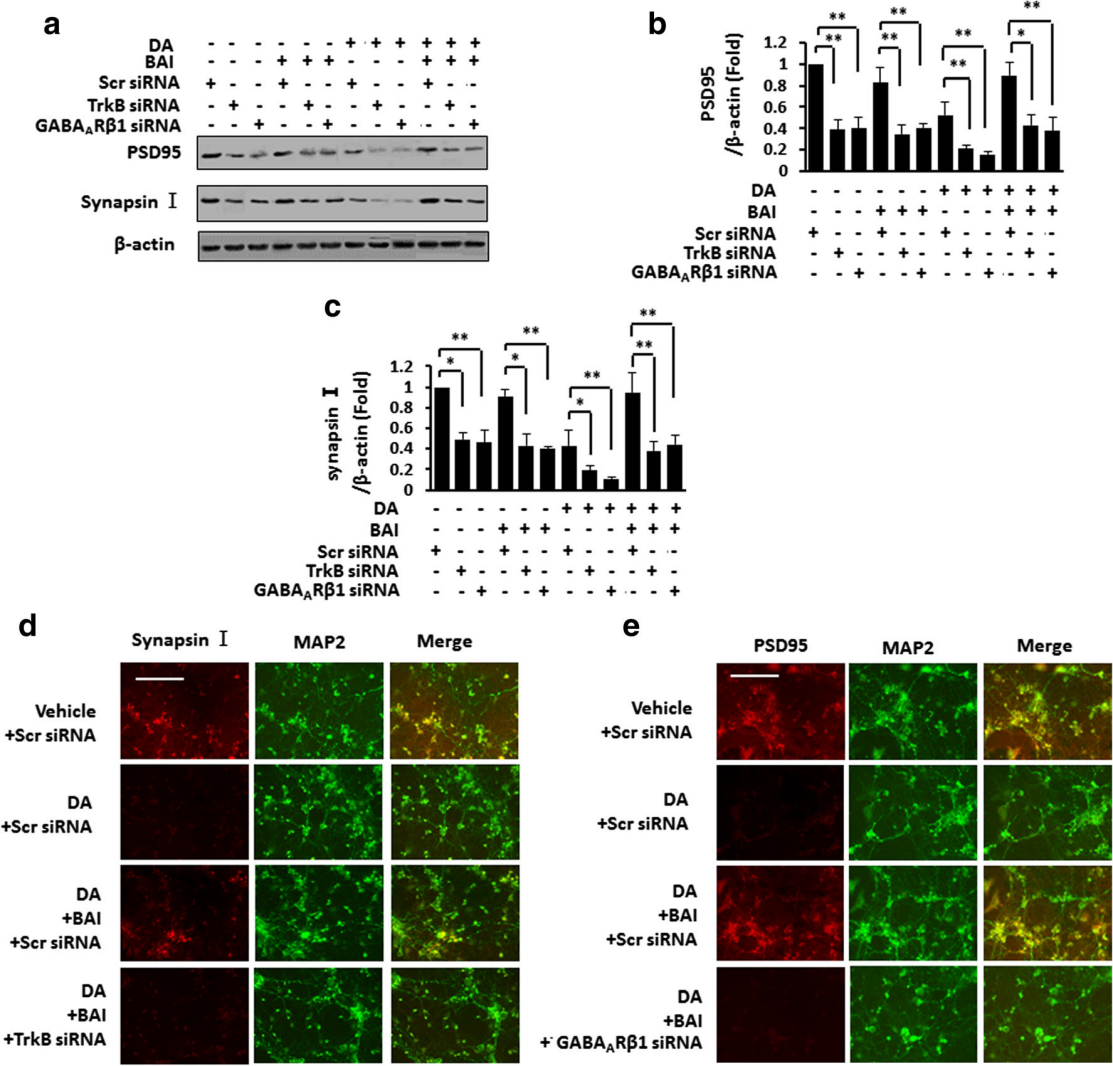
BAI prevents DA-induced impairment of synaptogenesis in vitro. **a** Immunoblot analysis of PHNs after TrkB/GABA_A_Rβ1 siRNA transfection stimulated with 10 μM DA in the presence of BAI (30 μM) using anti-PSD95/synapsin I and anti-β-actin antibodies and subsequent densitometry (**b**, **c**). **d**, **e** Double immunofluorescence staining of PHNs after TrkB/GABA_A_Rβ1 siRNA transfection stimulated with 10 μM DA in the presence of BAI (30 μM) using antibodies against PSD95 (**d**)/synapsin I (**e**) (red), MAP2 (green). *P < 0.05, **P < 0.01 vs. DA-treated PCNs. Scale bar, 25 μm. MRGD, merged image

We then examined whether BAI reversed the DA-induced synaptic loss via TrkB signaling. IB analysis indicated that PSD and synapsin I levels were decreased by DA treatment, which was reversed by the addition of BAI in PHNs. Additionally, the effect of BAI was inhibited by TrkB or GABA_A_Rβ1 silencing (Fig. 5a–c). Double IF staining shows that both PSD95 (Fig. 5d) and synapsin I (Fig. 5e) levels were significantly decreased in DA-treated PHNs and were recovered to normal levels by the administration of BAI, while the effect of BAI was blocked by either TrkB siRNA or GABA_A_Rβ1 siRNA. These data suggest that BAI affects DA.

### BAI does not induce toxicity in the hippocampus and cerebral cortex

**Fig. 6:**
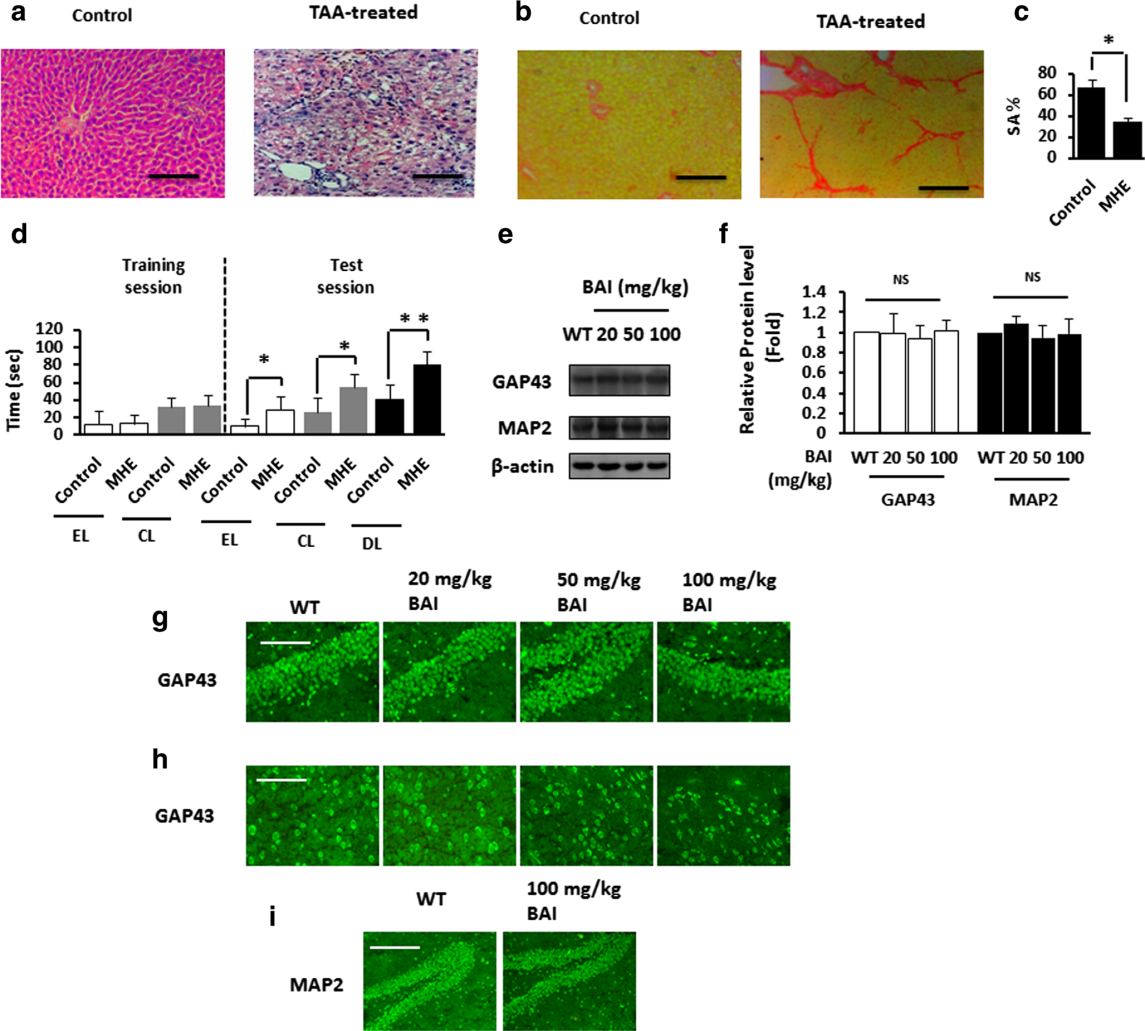
BAI does not induce toxicity in hippocampus and cerebral cortex. **a** HE staining of liver sections from TAA-treated rats. Scale bar, 50 μm. **b** Sirius red staining of liver sections from TAA-treated rats. Scale bar, 50 μm. **c** Spontaneous alternation percentages (SA%) in YM of normal rats and MHE rats. Data are shown as mean ± SD. *P < 0.05 vs. controls. **d** WFT results (EL entry latency, CL contacting latency, DL drinking latency) of normal rats and MHE rats. Data are shown as mean ± SD. *P < 0.05, **P < 0.01 vs. controls. **e** Immunoblot analysis of hippocampal lysates from WT rats treated with various concentrations of BAI (20, 50, 100 mg/kg) using anti-GAP43/MAP2 and anti-β-actin antibodies and subsequent densitometry (**f**). **g** Immunofluorescence staining of the hippocampus sections from WT rats treated with various concentrations of BAI (20, 50, 100 mg/kg) using antibodies against GAP43 (green). Scale bar, 50 μm. **h** Immunofluorescence staining of cortical sections from WT rats treated with various concentrations of BAI (20, 50, 100 mg/kg) using antibodies against GAP43 (green). Scale bar, 25 μm. **i** Immunofluorescence staining of hippocampal sections from WT rats treated with various concentrations of BAI (20, 50, 100 mg/kg) using antibodies against MAP2 (green). Scale bar, 100 μm. NS not significant

First, we successfully established the MHE rat model (Fig. 6a, b) and DA-injected rat model (Fig. 6c, d). Then, we evaluated the potential toxicity of orally administered BAI to the brain in vivo. Immunoblotting showed no significant difference in the expression levels of 43-kD growth-associated protein (GAP-43) and MAP2 between the control and the BAI groups (Fig. 6e, f). Furthermore, GAP-43 immunostaining revealed no obvious differences between the vehicle- and the BAI-treated rats in the distribution and intensity of immunofluorescence in the hippocampus (Fig. 6g) and cerebral cortex (Fig. 6h). Likewise, there were no significant differences between the vehicle- and the BAI-treated rats in the distribution and intensity of MAP2 immunofluorescence in the hippocampus (Fig. 6i). These findings indicate that BAI causes no obvious toxicity to the rat brain.

### BAI reverses the inactivation of the GABA_A_Rβ/TrkB signaling pathway in MHE rats and DA-treated rats

**Fig. 7:**
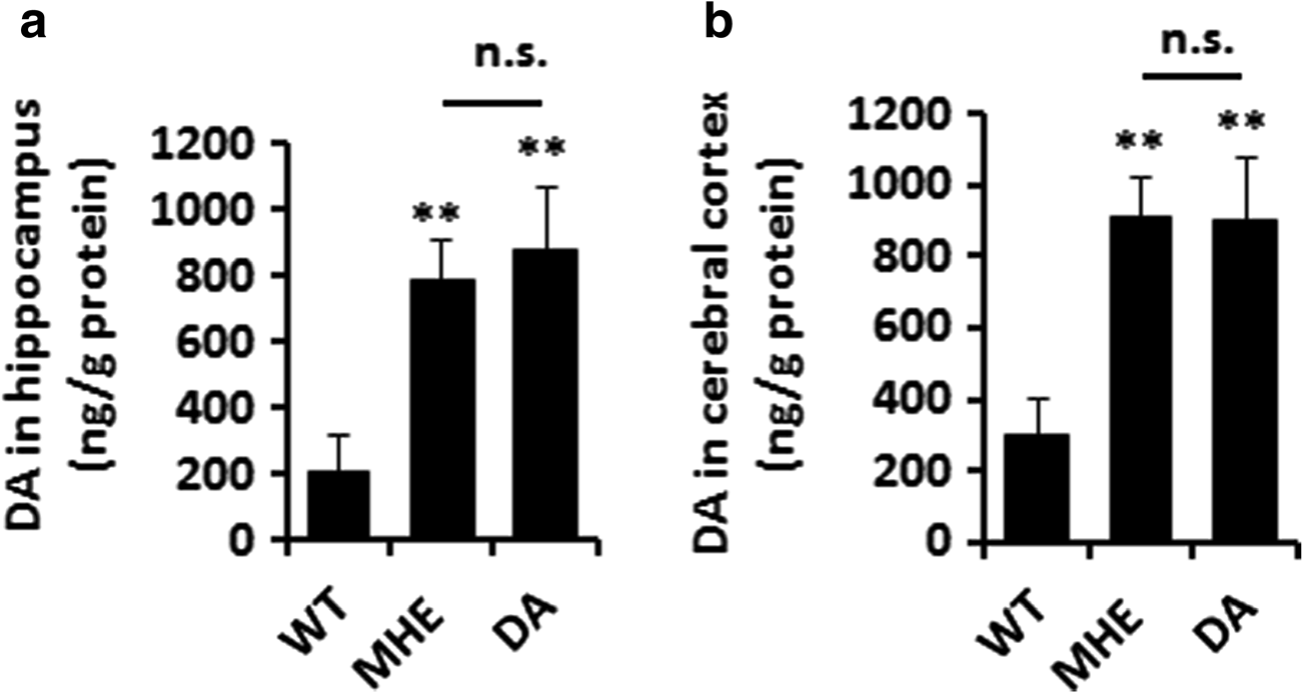
Concentration of intracranial DA in MHE and DA-treated rats. **a** Hippocampal homogenates from MHE and DA-treated rats were analyzed for DA concentration by high-performance liquid chromatography. **b** Cerebral cortex homogenates from MHE and DA-treated rats were analyzed for DA concentration by high-performance liquid chromatography

We examined whether the concentration of dopamine in the hippocampus and cortex of MHE rats was as high as that in DA-treated rats. We confirmed increased levels of DA in the hippocampus (Fig. 7a) and cerebral cortex (Fig. 7b) in both MHE rats and DA-treated rats compared with control rats, and the DA levels were as high in MHE rats as in DA-treated rats.

**Fig. 8:**
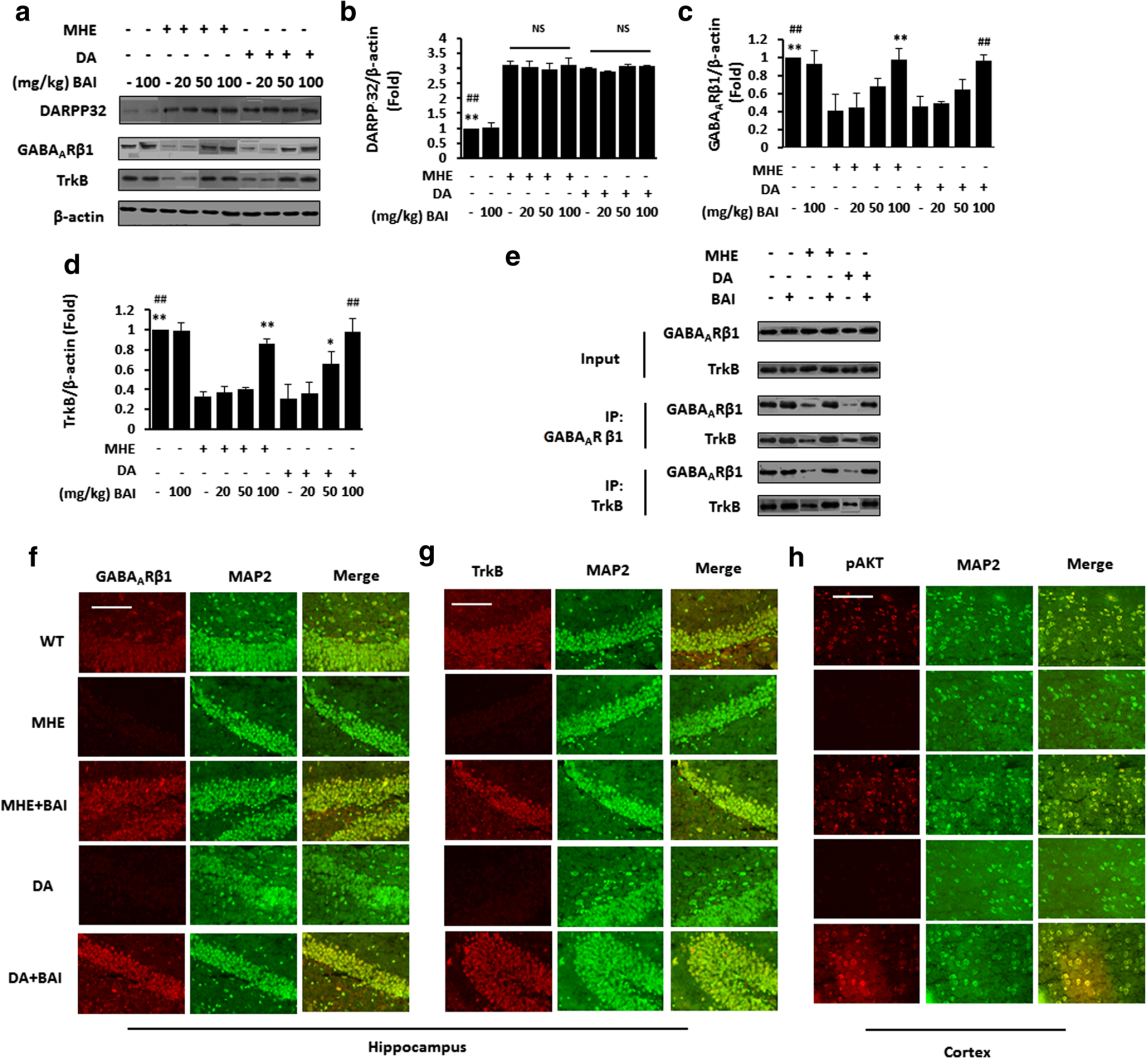
Effect of BAI on the DARPP32/GABA_A_R/TrkB signaling pathway in MHE rats and DA-treated rats. **a** Immunoblot analysis of hippocampal lysates from MHE and DA-treated rats administered various concentrations of BAI (20, 50, or 100 mg/kg) using anti-DARPP 32/GABA_A_Rβ1/TrkB and anti-β-actin antibodies and (**b**–**d**) subsequent densitometry. **e** Immunoprecipitation of hippocampal lysates from MHE and DA-treated rats administered BAI (100 mg/kg) with control IgG, anti-TrkB, or anti-GABA_A_Rβ1 antibodies. Complexes were immunoblotted with anti-TrkB or anti-GABA_A_Rβ1 antibodies. **f** Double immunofluorescence staining of hippocampal sections from MHE or DA-treated rats administered BAI (100 mg/kg) using antibodies against GABA_A_Rβ1 (red), MAP2 (green). Scale bar, 25 μm. **g** Double immunofluorescence staining of hippocampus sections from MHE or DA-treated rats administered BAI (100 mg/kg) using antibodies against TrkB (red), MAP2 (green). Scale bar, 100 μm. **h** Double immunofluorescence staining of cortical sections from MHE or DA-treated rats administered BAI (100 mg/kg) using antibodies against pAKT (red), MAP2 (green). Scale bar, 50 μm. *P < 0.05, **P < 0.01 vs. MHE rats; #*P* < 0.05, ##*P* < 0.01 vs. DA-treated rats. NS not significant. Scale bar, 25 μm. MRGD, merged image

Based on the previous result, we examined the effect of BAI on DARPP32 levels in vivo. IB analysis showed increased expression of DARPP32 in the hippocampus in MHE and DA-treated rats, which was unaffected by BAI administration (Fig. 8a, b).

Then, we examined the effect of BAI on the interaction of GABA_A_Rβ with TrkB in vivo. Decreased hippocampal GABA_A_Rβ (Fig. 8a, c) and TrkB (Fig. 8a, d) expression was found in MHE and DA-treated rats, and the administration of BAI increased the expression of GABA_A_Rβ and TrkB. Further co-immunoprecipitation experiments confirmed decreased interaction between GABA_A_Rβ and TrkB in MHE and DA-treated rats when GABA_A_Rβ and TrkB were immunoprecipitated in the cerebral cortex and hippocampus, and BAI administration improved the interaction (Fig. 8e), consistent with the Western blot results. Immunofluorescence staining showed that GABA_A_Rβ (Fig. 8f) and TrkB (Fig. 8g) were weakly expressed in the hippocampal neurons of MHE and DA-treated rats but strongly expressed after the administration of BAI. In the cerebral cortex, immunofluorescence revealed decreased expression of pAKT (Fig. 8h) in both MHE rats and DA-treated rats, and BAI ameliorated the decrease in pAKT expression. These results suggest that BAI does not affect the DA-induced activation of DARPP32 signaling and reverses the DA-mediated inactivation of the downstream GABA_A_Rβ/TrkB signaling pathway in MHE rats.

### BAI prevents the impairment of synaptogenesis in MHE rats and DA-treated rats

**Fig. 9:**
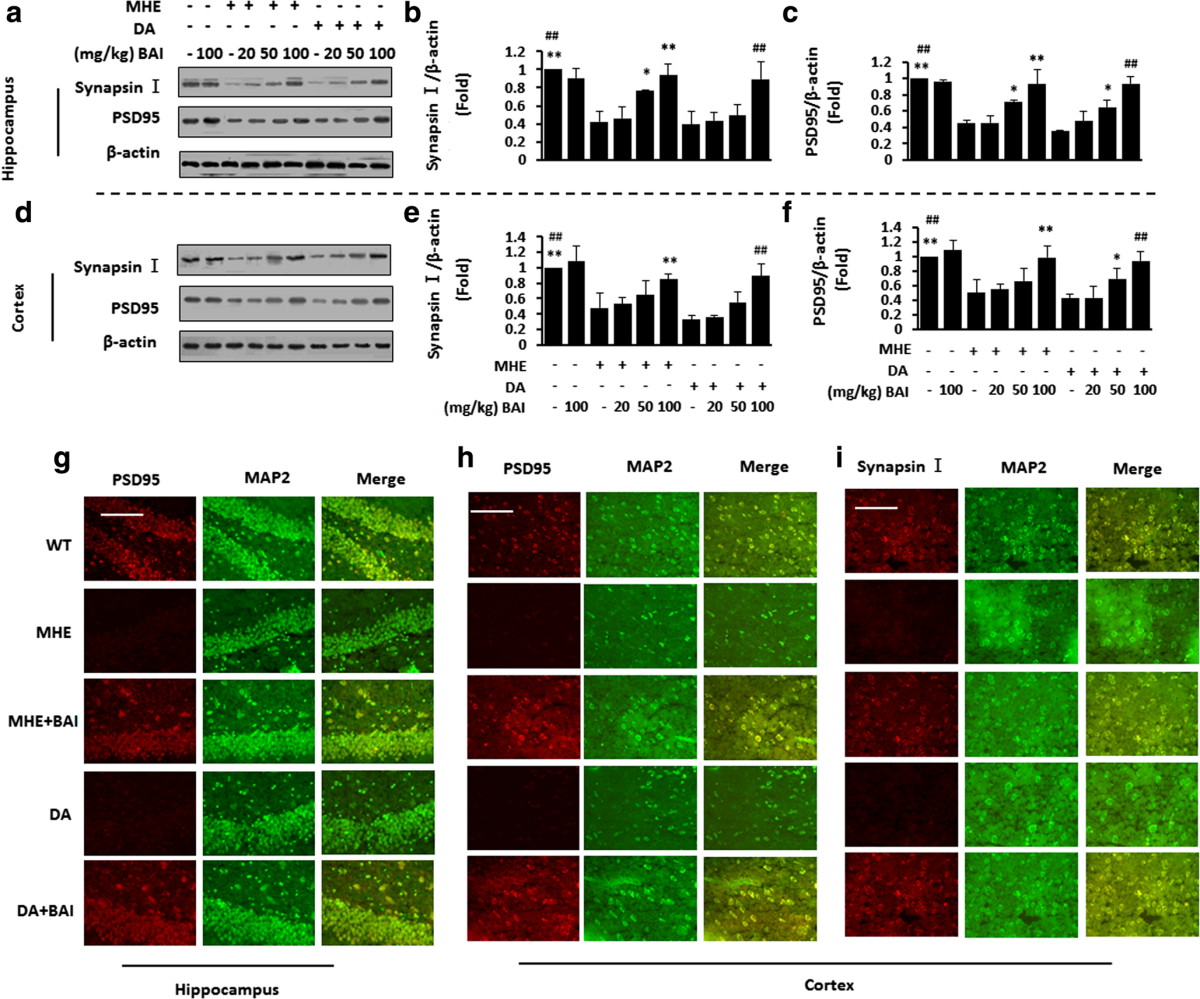
BAI prevents synaptic loss in MHE rats and DA-treated rats. **a**–**f** Immunoblot analysis and subsequent densitometry of hippocampal (**a**–**c**) and cortical (**d**–**f**) lysates from MHE or DA-treated rats administered various concentrations of BAI (20, 50, 100 mg/kg) using anti-PSD95/synapsin I and anti-β-actin antibodies. **g** Double immunofluorescence staining of hippocampal sections from MHE or DA-treated rats administered BAI (100 mg/kg) using antibodies against PSD95 (red), MAP2 (green). (**h**, **i**) Double immunofluorescence staining of cortical sections from MHE or DA-treated rats administered BAI (100 mg/kg) using antibodies against PSD95 (**h**)/synapsin I (**i**) (red), MAP2 (green). *P < 0.05, **P < 0.01 vs. MHE rats; #*P* < 0.05, ##*P* < 0.01 vs. DA-treated rats. Scale bar, 25 μm. MRGD, merged image

We further examined the effect of BAI on presynaptic markers (synapsin I) and postsynaptic markers (PSD95) in vivo. Hippocampal fractions from MHE and DA-treated rats showed significant decreases in PSD95/synapsin I expression, and BAI treatment improved the expression of synaptic markers (Fig. 9a–c). Significant decreases in the expression of PSD95/synapsin I were also found in the cortices of MHE and DA-treated rats, and BAI treatment reversed this decrease (Fig. 9d–f). IF staining of the hippocampus (Fig. 9g) and cortex (Fig. 9h) in MHE and DA-treated rats showed decreased expression of PSD95, and BAI treatment decreased the levels of both proteins. IF staining also showed decreased levels of synapsin I in the cortices of MHE and DA-treated rats, and BAI treatment increased the protein levels (Fig. 9i). These results suggest that the induction of synaptogenesis by BAI inhibits the DA-induced loss of synaptogenesis via the activation of GABA_A_Rβ/TrkB in MHE rats.

### BAI reverses the impairment of memory function in MHE rats and DA-treated rats

**Fig. 10:**
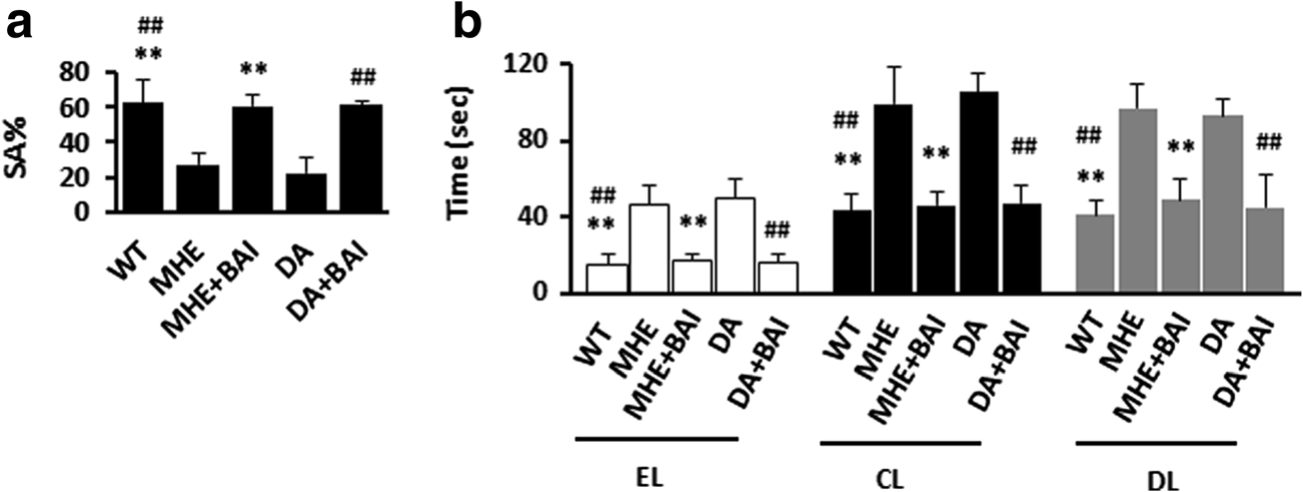
Effects of BAI on cognitive function in MHE rats and DA-treated rats. **a** Spontaneous alternation percentage (SA%) in YM of MHE rats or DA-treated rats administered 100 mg/kg BAI. **b** WFT results (EL entry latency, CL contacting latency, DL drinking latency) of MHE rats or DA-treated rats administered 100 mg/kg BAI. *P < 0.05, **P < 0.01 vs. MHE rats; #*P* < 0.05, ##*P* < 0.01 vs. DA-treated rats

Finally, we assessed whether BAI improved memory impairment in vivo. TAA-treated rats with no HE symptoms were subjected to behavioral tests (a YM and a WFT). In the YM, the decreases in SA% in MHE rats and DA-treated rats were reversed by the administration of BAI (Fig. 10a). In the WFT, the significant delays in EL, CL, and DL in MHE rats and DA-treated rats were recovered to the normal levels by BAI treatment (Fig. 10b). These findings indicate that BAI reverses DA-induced cognitive decline in MHE rats.

## Discussion

The GABA_A_ receptor, which belongs to the fast-acting ligand-gated ion channel superfamily, mediates most of the inhibitory synaptic transmissions in the central nervous system and mediates GABAergic inhibition in cortical network assemblies (Macdonald and Olsen 1994). The ionotropic GABA_A_ receptors are heteromeric pentamers composed of different subunits from seven classes (α1–α6, β1–β3, γ1–γ3, δ, ϵ, π, and ρ). The majority of native receptors contain two α subunits, two β subunits, and one γ subunit (McKernan and Whiting 1996). The role of GABA_A_ receptors (GABA_A_Rs) in conjunctive object-location learning remains to be established. In this respect, it is interesting to note a potential effect of several subunits of the GABA_A_ receptor in object recognition behavior (Pirker et al. 2000). Alterations in the GABAergic system, including changes in tonic inhibition, are likely to be involved in the cognitive deficits associated with schizophrenia (Damgaard et al. 2011). Many pharmacological aspects of the GABAergic neurotransmitter system have been elucidated, including its anxiolytic, anti-convulsive, and anesthetic activities (Brailowsky and García 1999). In addition, the involvement of the GABAergic neurotransmitter system in learning and memory is supported by a substantial body of evidence (Makkar et al. 2010). Only a handful of studies have attempted to determine how DA influences this intrastriatal GABAergic pathway. Most of these studies have focused on the DA’s actions at D_1_R. For example, D1 dopamine receptor stimulation was found to increase GABA release (Harsing and Zigmond 1997). However, D1 dopamine receptor agonists appear to be ineffective in modulating GABAergic synaptic potentials in dorsal neostriatal neurons, despite the fact that they inhibit GABAergic signaling in the nucleus accumbens (Nicola and Malenka 1998). At face value, the inability of D_1_R agonists to modulate GABAergic synaptic potentials in medium spiny neurons is surprising (Surmeier et al. 1995). Excess DA has been shown to modulate GABA_A_ receptors in the hippocampus and cerebral cortex. D1R receptors positively couple to DARPP32, resulting in the inactivation of GABA_A_R in MHE. The results of the present study suggest that the overstimulation of D1R by DA overload heightens the efficacy of DARPP-32. The occurrence of tonic GABA_A_ receptor inhibition is irrelevant to the expression of α1 and α4 subunits. These results suggest that at high levels of D_1_R stimulation, DARPP32 effectively blocks the expression of GABA_A_ receptor β1 and β3 subunits. The upregulation of D_1_R and downregulation of the β1 and β3 subunits of GABA_A_ receptors in MHE rats imply that both D1R and GABA_A_Rβ are involved in the underlying pathology of MHE.

The family of compounds with a flavonoid structure and binding affinity to GABA_A_ receptors, which includes BAI, has been shown to exert benzodiazepine (BZ)-like pharmacological actions with a wide spectrum of efficacies (Wang et al. 2005). A characteristic of GABA_A_ receptors is their capacity for modulation by classical BZs, such as diazepam (Rabow et al. 1995). Zhang et al. (2006) demonstrated that BAI passed through the blood–brain barrier and distributed within brain tissue, specifically in the hippocampus, striatum, cortex, and thalamus, although the exact mechanism was not reported. Several studies have shown that BAI prevents cognitive dysfunction and reduces the apoptosis of hippocampal pyramidal cells in global cerebral ischemia/reperfusion injury in rats and humans (Cheng et al. 2012). Moreover, BAI protects the central nervous system activity following seizure-induced brain injury (Cao et al. 2010; Liu et al. 2012), promotes neuronal differentiation (Li et al. 2011), and guards against degeneration and neuronal dysfunction (Tu et al. 2011). It is worth noting that BAI activates GABAergic signaling in global ischemia, which may be a mechanism underlying its neuroprotective effect (Dai et al. 2013). In this study, we observed a decrease in the expression of GABA_A_Rβs in the hippocampi of MHE and DA-treated rats and found that BAI exerted a beneficial effect on the expression of GABA_A_Rβ. Our results demonstrate that BAI mimics GABA and exerts neurotrophic actions by protecting cortical and hippocampal neurons from DA-induced toxicity and dendritic and synaptic loss via the dopaminergic suppression of GABA_A_R currents in vitro. In addition, our data showed that intranasal administration of BAI did not significantly alter the structures or expression levels of GAP-43 and MAP2 in hippocampal or primary cortical neurons, suggesting that oral administration of BAI may represent a safe and noninvasive therapeutic strategy for the treatment of MHE.

All of these findings suggest that BAI exerts its neurotrophic activity through GABA_A_Rβ. TrkB signaling plays a direct role in the formation and maintenance of synapses (Hiester et al. 2013), and TrkB stimulation plays critical roles in neuronal plasticity, survival, and neurogenesis (Diniz and Teixeira 2011; Zuccato and Cattaneo 2009). TrkB activation is required for multiple aspects of neuronal function, including neuronal survival, morphological change, and synaptic plasticity (Diniz and Teixeira 2011; Zuccato and Cattaneo 2009). In our study, the expression of synaptic markers was increased by BAI treatment, and the silencing of TrkB blocked the effect of BAI, indicating that BAI exerts a profound protective effect on synapses by activating TrkB in vitro. Rat models of MHE showed deficits in hippocampal LTP, which correlated with the impairment in the hippocampus-dependent memory. LTP is regarded as a cellular mechanism for learning and memory (Wen et al. 2013). Furthermore, synaptic loss appears to be the best pathologic correlate of dementia in HE (Schroeter et al. 2015).

Given the key roles that GABA_A_Rβ–TrkB signaling plays in learning and memory, we proposed that BAI could prevent memory decline in MHE rats. Tyrosine receptor kinase B (TrkB) signaling in neurons promotes the formation and postsynaptic localization of GABA_A_R clusters (Binder 2007; Kafitz et al. 1999) and regulates GABA_A_R functions (Elmariah et al. 2005; Lindholm et al. 1994) and responses (Pearce 1993; Pearce et al. 1995). Meanwhile, TrkB signaling cascade is functionally coupled with GABA_A_R (Squinto et al. 1991; Tanaka et al. 1997). We previously reported that BAI binds to GABA_A_Rβ and quickly induces TrkB dimerization, phosphorylation, and the activation of downstream AKT/synapsin I signaling pathways (Andero et al. 2011; Jang et al. 2010). The results of the present study demonstrate that the activation of GABA_A_Rβ is coupled with the upregulation of TrkB, and BAI displays neurotrophic actions by protecting PHNs and PCNs from DA-induced synaptic loss in vitro. These findings suggest that BAI simulates the physiological actions of the GABA_A_Rβ/TrkB signaling pathway and prevents synaptic dysfunction and cognitive deficits in a rodent model of MHE.

Based on these findings, we hypothesized that synaptic dysfunction is a major pathophysiological hallmark of MHE. It has been suggested that “synaptoprotective” therapy may have more clinical relevance than neuroprotective therapy for MHE (Zhang et al. 2006). One finding suggested that GABAergic transmission may be a critical step in the process of homeostatic plasticity, such that activity blockage reduces GABA release and therefore GABA_A_Rβ activation, which then triggers synaptic scaling (Wenner 2014). If this is true, we would predict that activity blockage and GABA_A_Rβ blockage would induce scaling via a common mechanism: a shift in the chloride reversal potential. Our study identified an essential role for BAI in the synaptic integration of hippocampal and cortical neurons and suggested an activity-dependent mechanism for synaptic contact. Like GABA, BAI promotes the expression of presynaptic markers (synaptotagmin and synapsin) and postsynaptic markers (PSD95 and spinophilin), leading to synaptogenesis. Furthermore, chronic exposure of the brain to a high concentration of BAI is sufficient to exert a protective effect in vivo.

**Fig. 11:**
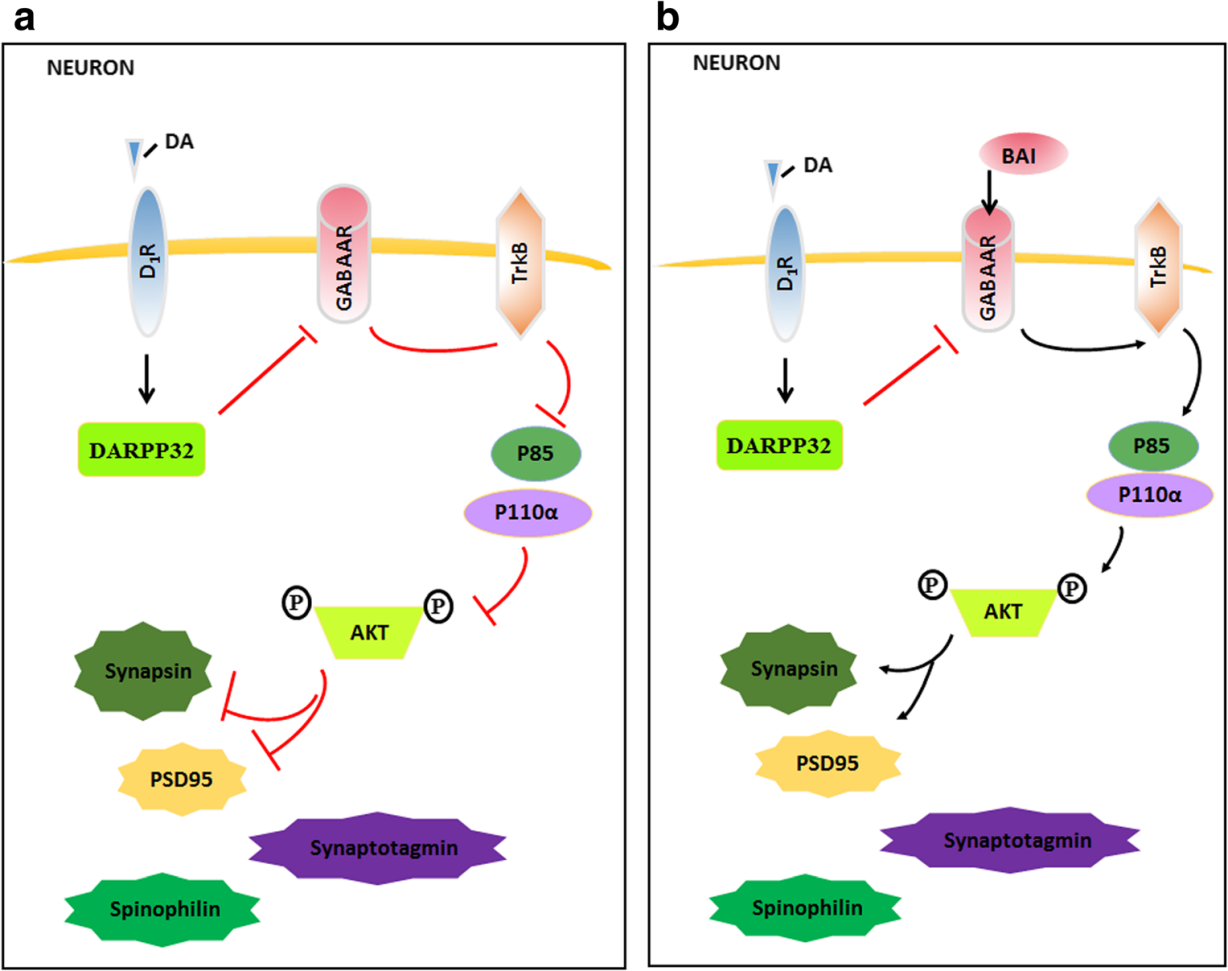
DA mediated the destruction of the synaptogenesis signaling pathway. **a** DA initiates its effects by binding to a dopamine receptor. Once activated, the DR recruits DARPP32, which inhibits the activation of GABA_A_Rβ, then destroys the TrkB signaling, and subsequently dephosphorylates AKT. Inactivated AKT leads to the downregulation of presynaptic markers (synaptotagmin and synapsin I) and postsynaptic markers (PSD95 and spinophilin), which induces synaptic loss. **b** Conversely, BAI is recruited to bind to GABA_A_Rβ1, which activates TrkB signaling and subsequently phosphorylates AKT. Activated AKT leads to the upregulation of presynaptic markers (synaptotagmin and synapsin I) and postsynaptic markers (PSD95 and spinophilin), which potentiates synaptic loss

In summary, this study demonstrated that the administration of BAI exerts a therapeutic effect in MHE rats. This effect can largely be attributed to the protective effect of BAI on synapses. The neuroprotective effects of BAI may be linked to the modulation of the interaction of GABA_A_Rβ and TrkB and the elevation of synaptic protein expression. Our study identified BAI as a novel synaptoprotective strategy for the treatment of MHE. Our data also showed that BAI regulates GABA_A_Rβ to activate the TrkB/AKT/synapse-related protein pathway. BAI can also promote the activation of GABA_A_Rβ, a likely mechanism by which BAI reverses DA-induced LTP impairments in MHE rats. Based on the results of the present study, BAI may serve as a useful drug for the treatment of patients with MHE; however, it is essential to further explore whether other modulators are involved in the neuroprotective effects of BAI against MHE (Fig. 11).
